# RNA Viral Metagenome of Whiteflies Leads to the Discovery and Characterization of a Whitefly-Transmitted Carlavirus in North America

**DOI:** 10.1371/journal.pone.0086748

**Published:** 2014-01-21

**Authors:** Karyna Rosario, Heather Capobianco, Terry Fei Fan Ng, Mya Breitbart, Jane E. Polston

**Affiliations:** 1 College of Marine Science, University of South Florida, Saint Petersburg, Florida, United States of America; 2 Department of Plant Pathology, University of Florida, Gainesville, Florida, United States of America; University of California Riverside, United States of America

## Abstract

Whiteflies from the *Bemisia tabaci* species complex have the ability to transmit a large number of plant viruses and are some of the most detrimental pests in agriculture. Although whiteflies are known to transmit both DNA and RNA viruses, most of the diversity has been recorded for the former, specifically for the *Begomovirus* genus. This study investigated the total diversity of DNA and RNA viruses found in whiteflies collected from a single site in Florida to evaluate if there are additional, previously undetected viral types within the *B. tabaci* vector. Metagenomic analysis of viral DNA extracted from the whiteflies only resulted in the detection of begomoviruses. In contrast, whiteflies contained sequences similar to RNA viruses from divergent groups, with a diversity that extends beyond currently described viruses. The metagenomic analysis of whiteflies also led to the first report of a whitefly-transmitted RNA virus similar to Cowpea mild mottle virus (CpMMV Florida) (genus *Carlavirus*) in North America. Further investigation resulted in the detection of CpMMV Florida in native and cultivated plants growing near the original field site of whitefly collection and determination of its experimental host range. Analysis of complete CpMMV Florida genomes recovered from whiteflies and plants suggests that the current classification criteria for carlaviruses need to be reevaluated. Overall, metagenomic analysis supports that DNA plant viruses carried by *B. tabaci* are dominated by begomoviruses, whereas significantly less is known about RNA viruses present in this damaging insect vector.

## Introduction

The majority of vectored plant viruses are transmitted by hemipteran insects, whose piercing-sucking mouthparts allow efficient transmission [1]. Whiteflies (*Aleyrodidae*), in particular the *Bemisia tabaci* species complex, are among the most detrimental insect vectors causing considerable economic losses to multiple agricultural industries [2], [3]. Whiteflies damage crops directly through feeding, which can weaken plants and elicit undesirable plant responses [4], and through depositing excreta that favors sooty mold production. In addition, whiteflies indirectly damage crops by transmitting pathogenic viruses [2], [3], [5]. Viruses are responsible for almost half of the emerging diseases affecting plants and whitefly-transmitted viruses are some of the most devastating agents affecting cash crops [6].

Among the large diversity of viral types known to infect plants, only DNA viruses belonging to the genus *Begomovirus* (*Geminiviridae*) and a small diversity of RNA viruses have been associated with whiteflies. Whiteflies are known to transmit more than 280 begomovirus species [5], [7] and, to our knowledge, no other DNA viral groups have been detected in whiteflies. The emergence of begomoviruses as important pathogens is closely associated with the increased prevalence of highly polyphagous whitefly species [5]. Whiteflies feed on a large number of cultivated and native plant species and thus may provide the opportunity to transmit viruses among a variety of hosts, including wild and cultivated vegetation [3], [8], [9]. The ability of whiteflies to transmit begomoviruses into diverse hosts, as well as the high potential for co-infection and recombination opportunities, may have contributed to the emergence of the genus *Begomovirus* as the group of plant viruses with the largest number of recognized species [5], [7]. In contrast to the species-rich, genus-specific association of whiteflies with begomoviruses, a low species diversity of RNA viruses is known to be vectored by whiteflies. There are four genera of RNA viruses (each with fewer than 15 species) known to be transmitted by whiteflies, namely: *Crinivirus* (*Closteoviridae*; 12 species), *Carlavirus* (*Betaflexiviridae*; 1 species), *Ipomovirus* (*Potyviridae*; 4 species), and *Torradovirus* (*Secoviridae*; 4 species) [5].

It is possible that the diversity of whitefly-transmitted viruses reported to date does not accurately represent the total complement of viruses in this insect vector. Viral types known to be carried by *B. tabaci* may instead reflect methodological limitations that are only capable of detecting close relatives of known vector-transmitted viruses (e.g., PCR with degenerate primers designed based on known sequences). In addition, agricultural surveillance efforts typically focus on viral species that negatively affect economically important crops. Therefore it is likely that viruses present in native vegetation that do not show any impact on agricultural crops in the area will be overlooked. Since viruses found in native vegetation may emerge as serious pathogens for crops and asymptomatic hosts may facilitate virus spread by serving as reservoirs [10]–[12], there is a critical need to investigate the total community of DNA and RNA viruses associated with whiteflies in a given area. This endeavor can be accomplished by applying the vector-enabled metagenomics approach (VEM; where viruses are purified and sequenced directly from insect vectors) using whiteflies. The main advantage of VEM is that it allows the detection of viruses carried by insect vectors without *a priori* knowledge of the plant pathogens present in a given area [13]. Moreover, the VEM approach does not depend on the collection of foliar tissue exhibiting virus-like infection symptoms to detect viruses present in an area, circumventing limitations associated with sampling individual plants and enabling the identification of asymptomatic infections. VEM using a small sequencing effort has been successfully implemented to identify whitefly-transmitted begomoviruses infecting both commercial crops and native vegetation [13].

In an effort to shed light on the total diversity of viruses carried by whiteflies, this study incorporated high-throughput sequencing into the VEM approach to detect DNA and RNA viruses in *B. tabaci* specimens collected from an experimental field site in Florida. Begomoviruses were the only DNA plant viruses detected, whereas known and novel RNA viruses from different families were found in whiteflies from this single field site. Furthermore, sequencing efforts resulted in the detection and first report of a whitefly-transmitted carlavirus most similar to Cowpea mild mottle virus (CpMMV) in North America. Although the CpMMV Florida isolate was originally detected in whiteflies, it was subsequently identified in wild and cultivated plants from the same area and its host range was experimentally determined. Analysis of the CpMMV Florida genome suggests that the current classification criteria for carlaviruses need to be reevaluated.

## Materials and Methods

### Whiteflies: Sample collection, processing, and metagenomic sequencing

The *B. tabaci* specimens used for viral metagenomics were collected in an experimental field in Citra, Florida (29°24′N 82°06′W) in August 2007 as previously described [13]. Briefly, adult whiteflies were collected from soybean and volunteer watermelon plants using a battery-operated vacuum. The whiteflies were manually inspected using a Nikon model C-DSD115 stereoscope and debris and other insects were removed before storing at −80°C. A subset of the whiteflies were used in a pilot study investigating DNA viruses [13], while the remainder were processed for the present study as described below.

Virus particles were partially purified from the whiteflies before nucleic acid extraction and sequencing. For this purpose, approximately 250 whiteflies were homogenized in SM Buffer (50 mM Tris·HCl, 10 mM MgSO_4_, 0.1 M NaCl, pH 7.5) using a bead-beater (BioSpec) with 1.0 mm glass beads (Research Products International) for 1 min. Cells were removed from homogenates by centrifuging at 10,000 *xg* for 10 min and filtering the supernatant through a 0.22 µm Sterivex filter (Millipore). Virus particles present in the filtrate were treated with 0.2 volumes of chloroform, followed by DNase I (2.5 U/µl) and RNase A (0.25 U/µl) treatment at 37°C for 3 hrs to remove non-encapsidated nucleic acids.

DNA and RNA were simultaneously extracted from purified virus particles using the All Prep DNA/RNA Mini Kit (Qiagen) following manufacturer's instructions and sequenced individually. For the RNA fraction, the ‘on-column DNase digestion’ step was used to minimize DNA carryover. The extracted DNA fraction was amplified using the GenomiPhi V2 DNA Amplification Kit (GE Healthcare) followed by further amplification and fragmentation using the GenomePlex Whole Genome Amplification (WGA) Kit (Sigma-Aldrich). The RNA fraction was amplified using the TransPlex Whole Transcriptome Amplification (WTA) Kit (Sigma-Aldrich). WGA- and WTA-amplified nucleic acids were used for next-generation sequencing using a single lane of a Genome Analyzer IIx System (Illumina) by multiplexing.

### Metagenomic data analysis

WGA and WTA adapter sequences as well as multiplexing barcodes were removed from the DNA and RNA sequence libraries, respectively, using the TagCleaner server (http://edwards.sdsu.edu/cgi-bin/tagcleaner/tc.cgi) [14]. Trimmed sequences from both DNA and RNA libraries are publicly available on the Metavir website (http://metavir-meb.univ-bpclermont.fr/) under the project ‘Whiteflies_Citra_2007’. Sequences (1.4 million from the DNA library and 2.1 million from the RNA library) were then assembled with a minimum identity of 95% over 25 bp using the Geneious software package (Biomatters). Contigs over 80 bp in length were compared against the GenBank non-redundant database using either BLASTn (DNA library) or BLASTx (RNA library) with an e-value cut-off of E<0.001 in June 2011 [15]. BLAST results were summarized and inspected using the Metagenome Analyzer (MEGAN4) software [16] to identify viral sequences. The top viral match for each contig was accepted only if the score for the top virus hit was at least 10% higher than the next best hit; otherwise, the contig was annotated as “unassigned”. In most cases where the BLAST scores were within 10% of each other, the viral matches belonged to the same genus and thus the genus was identified.

### CpMMV Florida isolate genome completion

The majority of contigs from the RNA library with significant matches to viral sequences were similar to the carlavirus CpMMV. To sequence the full genome of this virus, contigs with similarities to CpMMV were organized based on the genomic position sharing similarity with a CpMMV reference genome from Africa (NC014730). Primer pairs were designed to bridge the gaps between contigs and primer pairs that spanned the entire genome were used to complete the genome (Table S1). cDNA for PCR reactions was produced from RNA extracted from purified virus particles using a SuperScript III First-Strand Synthesis System kit (Invitrogen). All PCR reactions contained 1 µl cDNA, 1 U Apex Red Taq Polymerase (Genesee), 1× NH_4_ buffer, 1.5 mM MgCl_2_, and 0.5 µM of each primer. Amplification was performed with an initial denaturation at 94°C for 5 min followed by 35 cycles of 94°C for 45 sec, 50°C for 45 sec (incrementally decreasing the temperature by 0.1°C each cycle), 72°C for 1.5 min, followed by a final extension at 72°C for 8 min. The 5′ end of the genome was completed using gene-specific primers with the 5′ RACE System Kit (Invitrogen) according to manufacturer's instructions (Table S1). All PCR products were cloned using the TOPO TA system (Invitrogen) and Sanger sequenced. PCR product sequences were assembled using Sequencher 4.7 (Gene Codes) and the complete genome was annotated using SeqBuilder (DNASTAR). Each region of the genome had at least 4× sequence coverage.

### Survey and isolation of CpMMV from wild and cultivated vegetation

To investigate the presence of the carlavirus CpMMV in the vegetation, 90 plants were surveyed in the summer of 2011 from an area around the field site in Citra, FL where whiteflies were originally collected. Leaves exhibiting a variety of viral infection symptoms (e.g., mottling, leaf curling) were collected from plants belonging to the *Fabaceae* family which are known hosts of CpMMV, namely peanuts (*Arachis hypogaea* L.; n = 71), hairy indigo (*Indigofera hirsuta* L.; n = 14), and dixie ticktrefoil (*Desmodium tortuosum* (Sw.) DC.; n = 5). All plant tissues were tested for the presence of CpMMV using a CpMMV-specific ELISA Reagent Set (Neogen Europe Ltd) in accordance with manufacturer's protocols. Samples were considered positive when their absorbance values were greater than the mean of the negative controls plus three standard deviations. Positive samples were verified through a degenerate carlavirus RT-PCR assay targeting part of the capsid protein (CP) and 3′end poly-A tail of these RNA genomes [17]. Briefly, RNA was extracted from plant tissues using TRI Reagent following manufacturer's protocols (Ambion Inc.). Reverse transcription was performed using ImProm-II™ Reverse Transcriptase (Promega) with the oligo-d(T21) primer according to manufacturer's protocols. The cDNA was used for PCR with the Carla-CP (5′GGBYTNGGBGTNCCNACNGA3′) and oligo-dT (21) primers under the following conditions: 0.5 ul cDNA, Taq DNA Polymerase (New England Biolabs), 1× standard Taq (Mg-free) buffer, 3.0 mM MgCl_2_, and 1 µM spermidine. Amplification was performed with an initial denaturation at 94°C for 5 min, followed by 35 cycles of 94°C for 1 min, 50°C for 1 min, and 72°C for 1 min ending with a final extension at 72°C for 5 min.

One *D. tortuosum* plant sample that tested positive for CpMMV by ELISA was used to establish a culture and obtain inoculum for transmission and host range determination experiments. The sample of *D. tortuosum* was mechanically inoculated to *Chenopodium quinoa* L., which is an established local lesion host for CpMMV [18], using a 1∶5 dilution of tissue to phosphate buffer (100 mM K_2_HPO_4_, 100 mM Na_2_HPO_4_, 10 mM Na_2_SO_3_, pH 7.4). Eight days later, chlorotic local lesions were observed on inoculated leaves of *C. quinoa*. Four of these lesions were removed and individually mechanically inoculated to primary leaves of common bean (*Phaseolus vulgaris* L. ‘Topcrop’) which is a known systemic host of some CpMMV isolates [19]–[22]. Three bean plants exhibited virus-like symptoms from this inoculation and tested positive for CpMMV by ELISA and RT-PCR.

### Whitefly transmission of isolated CpMMV

Viral isolates from each of the three infected bean plants were transmitted to new bean plants using *B. tabaci* (Mediterranean/Asia Minor/Africa clade, formerly known as *B. tabaci* Biotype B). For this purpose, infected beans were placed in separate cages in different growth rooms for acquisition and transmission. Transmissions were performed at different times throughout the day to prevent contamination through whitefly carryover between rooms. Non-viruliferous whiteflies were placed on each infected bean and given an acquisition access period of 20 min. Whiteflies were then transferred to three healthy beans and given an inoculation access period of 4 hrs. Transmission was terminated using insecticidal soap (20 ml/L Safer Soap®) and Imidacloprid (0.2% active ingredient formulation, applied as a 30 ml per plant drench). The presence of CpMMV was confirmed in all three whitefly-inoculated beans by RT-PCR. The CpMMV genome was sequenced from each of these bean plants through PCR using the same primers used to sequence the CpMMV genome from whiteflies (Table S1).

### Experimental CpMMV Florida host range

A variety of hosts were selected for experimental infectivity assays based on previously reported hosts for isolates of CpMMV (Table 1) [20]. Bean leaf tissue infected with an isolate of CpMMV from *D. tortuosum* was collected 19 days post inoculation, frozen and used as the inoculum source for all inoculations. Three to four experimental host species were tested at a time. Five to twenty plants of each species were mechanically inoculated at the first true leaf stage. At the same time, three to five plants of each test species were mock-inoculated to serve as negative controls and three to five common bean plants were inoculated to serve as positive controls for the quality of the inoculum. Plants were visually assessed daily and systemic symptoms were recorded at 14 days post inoculation. Plants were then sampled and tested for the presence of CpMMV by ELISA. Inconclusive results based on ELISA were further tested by RT-PCR.

**Table 1:** Responses observed in a range of selected host plants mechanically inoculated with the Cowpea mild mottle virus Florida isolate.

Family	Species	Cultivar	No. Infected/No.Inoculated^1^	Local Symptoms/Systemic Symptoms^2^
Amaranthaceae	*Gomphrena globosa* L.	Strawberry Fields	0/10	NS/NS
Chenopodeaceae	*Chenopodium giganteum* D. Don	n/a	0/5	NS/NS
**Chenopodeaceae**	***Chenopodium quinoa*** ** Willd.**	**n/a**	**8/10**	**CLL/NS**
Cucurbitaceae	*Cucumis sativus* L.	Straight Eight	0/10	NS/NS
**Fabaceae**	***Arachis hypogaea*** ** L.**	**GA Green**	**10/10**	**NS/NS**
**Fabaceae**	***Glycine max*** ** (L.) Merr.**	**Round-up Ready**	**10/10**	**NS/VC, LD, Mo**
**Fabaceae**	***Lens culinaris*** ** Medik.**	**Green Lentil**	**7/10**	**NS/NS**
**Fabaceae**	***Phaseolus coccineus*** ** L.**	**Scarlet Runner**	**10/10**	**NS/NS**
**Fabaceae**	***Phaseolus lunatus*** ** L.**	**Fordhook No. 242**	**10/10**	**NS/NS**
**Fabaceae**	***Phaseolus vulgaris*** ** L.**	**Topcrop**	**9/10**	**NS/Mo, R**
**Fabaceae**	***Pisum sativum*** ** L.**	**Lincoln**	**8/10**	**NS/NS**
**Fabaceae**	***Vigna radiata*** ** (L.) R. Wilczek**	**Mung bean**	**10/10**	**NS/NS**
**Fabaceae**	***Vigna unguiculata*** ** (L.) Walp.**	**CA Blackeye No. 5**	**10/10**	**RV, RLL/LD, mMo**
Solanaceae	*Capsicum annuum* L.	California Wonder	0/10	NS/NS
Solanaceae	*Datura stramonium* L.	n/a	0/10	NS/NS
Solanaceae	*Nicotiana glutinosa* L.	n/a	0/10	NS/NS
Solanaceae	*Solanum lycopersicum* L.	FL 7316	0/20	NS/NS
Solanaceae	*Solanum lycopersicum* L.	Sweetheart	0/20	NS/NS

^1^ Number of infected plants/number of plants inoculated. The number of infected plants was determined by ELISA.

^2^ Abbreviations used: NS - no symptoms, Mo – mottle, R – rugose, LD – leaf deformation, mMo – mild mottle, VC – veinal chlorosis, RV – red vein, RLL – red local lesions, CLL – chlorotic local lesions.

Infected species as determined by visible symptoms and/or ELISA are highlighted in boldface.

### CpMMV Florida genome, pairwise comparisons, and phylogenetic analysis

The CpMMV Florida genomes sequenced from whiteflies and bean plants as well as their predicted protein sequences were compared against known members of the *Carlavirus* genus. Predicted protein sequences were compared against the Pfam database [23] to identify conserved motifs. For all pairwise comparisons, alignments were performed using the MUSCLE algorithm [24] implemented in MEGA5 [25]. Pairwise distances were calculated in MEGA5 using p-distance and pairwise deletion of gaps. For phylogenetic analysis of the capsid protein, alignments were optimized using the PRALINE server [26] with default settings. A maximum likelihood tree was constructed using the PhyML online server [27] with the (LG+I+G+F) model chosen as the best-fit substitution model according to ProtTest [28]. The approximate likelihood ratio test (aLRT) was used to assess branch support [29].

## Results

### Viruses identified in whiteflies

VEM revealed a diversity of DNA and RNA plant viruses present in whiteflies collected from a single site in Citra, Florida. Viral sequences in the DNA library displayed high levels of similarity to previously described viruses, enabling their identification through BLASTn searches, while RNA sequences had to be identified through BLASTx due to limited similarities to known sequences. Viral contigs (n = 259) in the DNA library were dominated by begomoviruses (*Geminiviridae*; 97.3%), the majority of which shared >88% nucleotide identity with their top match in the database (Table S2). Note that short reads hinder any definitive classification of begomovirus species or strains; therefore, Table S2 only provides an overview of potential begomovirus types detected in whiteflies. In addition to begomoviruses, five contigs were most similar to novel begomovirus-associated satellites, Whitefly VEM Satellites, discovered in a nearby field [13]. Only two contigs were not related to plant viruses, including a single-stranded DNA bacteriophage and a human virus.

Although fewer viral contigs were recovered from the RNA library (n = 64) compared to the DNA library, the RNA sequences encompassed a broader viral diversity at the family level. The viral sequences identified in the RNA library had similarities to viruses from at least five different families (*Betaflexiviridae*, *Closteroviridae*, *Bunyaviridae*, *Bromoviridae*, *Virgaviridae*), three of which (*Bunyaviridae*, *Bromoviridae*, *Virgaviridae*) have not been detected in whiteflies previously (Table 2). Most of the identified RNA viruses were similar to plant viruses and contigs similar to the carlavirus CpMMV dominated the viral sequences. Viral sequences similar to plant viruses known to be transmitted by whiteflies, including criniviruses and CpMMV, had high amino acid identities (up to 100%) with their top match in the database. In contrast to the DNA library, many of the RNA viral contigs (33%) were highly divergent from known species since they shared less than 45% amino acid identity with their top match in the database. Several contigs had low identities to double-stranded RNA viruses, Circulifer tenellus virus 1 and Spissistilus festinus virus 1, recently discovered in plant-feeding hemipteran pests; however, it remains unknown whether these viruses replicate in insect cells or those of associated microorganisms [30]. Only three contigs had similarities to viruses that infect hosts other than plants or insects, including diatoms (Rhizosolenia setigera RNA virus) and humans (Uukuniemi virus and Armero virus). However, due to low amino acid identities, it is possible that these sequences represent novel plant or whitefly viruses.

**Table 2:** Plant or insect RNA viruses identified in whiteflies and amino acid (aa) identity ranges.

Virus match*	Hits	Contig length (nt)	AA identity range (%)	Genus	Family	Significance†	Ref.
**Cowpea mild mottle virus**	27	86–355	50–100	Carlavirus	Betaflexiviridae	N	
Unassigned carlavirus	9	84–284	64–100	Carlavirus	Betaflexiviridae	N	
**Lettuce chlorosis virus**	6	83–239	93–100	Crinivirus	Closteroviridae	N	
Circulifer tenellus virus 1	4	286–933	30–35	Unclassified	Unclassified	U	
Rice grassy stunt virus	2	391–579	23	Tenuivirus	Unclassified	U	
Spissistilus festinus virus 1	2	189–404	43	Unclassified	Unclassified	U	
Unassigned phlebovirus	2	685–931	24–43	Phlebovirus	Bunyaviridae	U	
Unassigned plant RNA virus	1	620	38–40	-	-	U	
Armero virus**	1	233	35	Tentative Phlebovirus	Bunyaviridae	U	
Unassigned bromovirus	1	299	37	Bromovirus	Bromoviridae	U	
Unassigned Ilarvirus subgroup 1	1	317	43	Ilarvirus	Bromoviridae	U	
Rhizosolenia setigera RNA virus**	1	328	39	Bacillariornavirus	Unclassified	U	
Rice stripe virus	1	734	32	Tenuivirus	Unassigned	U	
Tobacco mild green mosaic virus	1	749	35	Tobamovirus	Virgaviridae	U	
**Tomato chlorosis virus**	1	89	100	Crinivirus	Closteroviridae	K	[40]
Unassigned cilevirus	2	261–1159	22–38	Cilevirus	Unclassified	U	
Unassigned tobamovirus	1	384	33	Tobamovirus	Virgaviridae	U	
Uukuniemi virus**	1	313	28	Phlebovirus	Bunyaviridae	U	

Unassigned virus groups refer to contigs that had significant matches to more than one virus with BLASTn scores within 10% of each other. Multiple matches make it impossible to assign these partial sequences. Viruses found in hosts other than plants or insects are identified (**). Viruses highlighted in boldface are known to be whitefly – transmitted.

K = known virus, and known to occur in Florida at this site, N = known virus, but not previously reported from Florida, U = most likely a new, uncharacterized virus.

### Isolation and experimental host range determination of CpMMV Florida

Since CpMMV-like sequences were abundant in whiteflies and this virus had never been reported in North America, a survey of 90 symptomatic plants from three different species using ELISA with a CpMMV antibody was conducted in the same crop field four years after the whitefly collection. Thirty-eight percent of *A. hypogea* (n = 71), 36% of *I. hirsuta* (n = 14), and 100% of *D. tortuosum* (n = 5) plants tested positive for CpMMV. Note that infection symptoms observed in the field may not have been caused by CpMMV. Since all of the samples of *D. tortuosum* tested positive for CpMMV, an infected seedling from this species was used to establish a culture and obtain virus inoculum. This plant was used to mechanically infect *C. quinoa* and local lesions were subsequently inoculated into common bean plants (*P. vulgaris*). To confirm infection by CpMMV, the common bean plants were tested by ELISA and a degenerate carlavirus RT-PCR assay. Once the presence of CpMMV was confirmed, the CpMMV Florida isolate was successfully transmitted to three common bean plants using whiteflies. All three plants exposed to CpMMV-bearing whiteflies exhibited mild mottling symptoms and were verified as infected with CpMMV based on ELISA and RT-PCR.

To determine host range, a CpMMV isolate from a common bean plant infected through whitefly transmission was used to inoculate 18 species of experimental hosts belonging to five different families (*Amaranthaceae*, *Chenopodeaceae*, *Cucurbitaceae*, *Fabaceae*, *Solanaceae*). Ten of the 18 species tested were successfully infected by the CpMMV Florida isolate, all of which belonged to the *Chenopodeaceae* and *Fabaceae* families (Table 1). Six of the ten infected species did not show any visible symptoms of infection and had the same appearance as the negative controls; only four species exhibited local or systemic symptoms of infection. *C. quinoa* showed local chlorotic lesions on inoculated leaves, *Vigna unguiculata* exhibited both local and systemic symptoms, whereas *Glycine max* and *Pisum sativum* only displayed systemic symptoms (Table 1).

### CpMMV Florida genome

**Figure 1:**
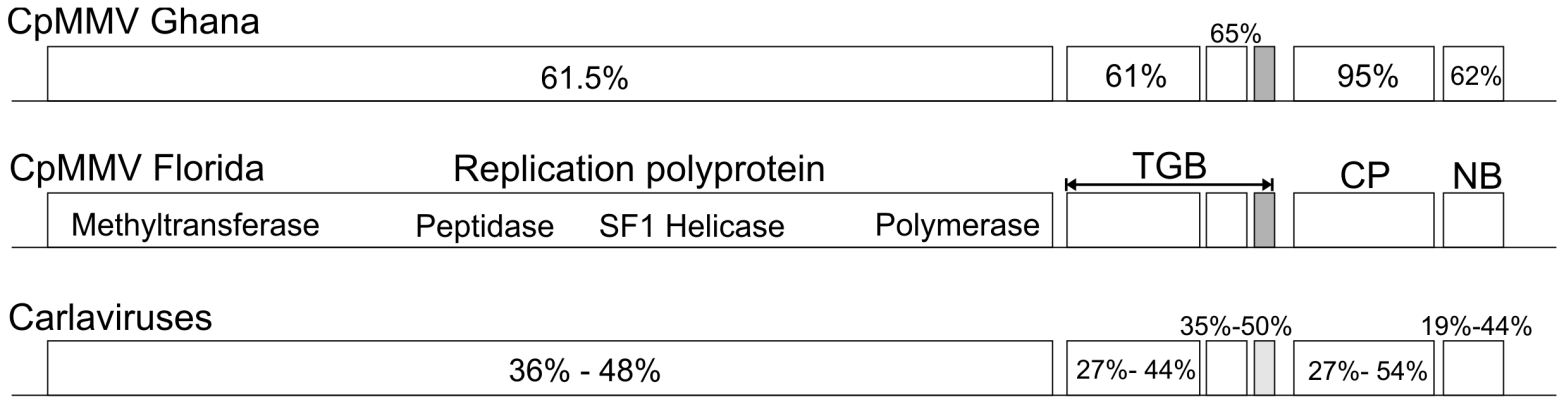
Schematic genome organization of Cowpea mild mottle virus (CpMMV) isolates identified in Ghana (top) and Florida (middle) as well as the general genome organization observed in carlavirus species (bottom; exact species shown in phylogenetic tree in Figure 2). Each block represents identified open reading frames (ORFs) in these genomes and the encoded protein names are given on top of the CpMMV Florida panel, including the replication polyprotein, triple gene block (TGB), capsid (CP), and nucleic acid binding (NB) protein. The four different domains within the replication polyprotein exhibiting viral methyltransferase, peptidase, superfamily one (SF1) helicase, and RNA-dependent RNA polymerase (polymerase) motifs are indicated. Percentage values in the CpMMV Ghana genome schematic represent amino acid pairwise identities among individual ORFs of CpMMV Ghana and CpMMV Florida. Percentages in the Carlaviruses genome schematic represent amino acid pairwise identity ranges observed between different carlavirus species and CpMMV Florida. ORFs highlighted in grey represent ORFs without a standard start codon (a lighter grey in the carlaviruses panel indicates that some carlavirus species have a standard start codon while others do not).

**Figure 2:**
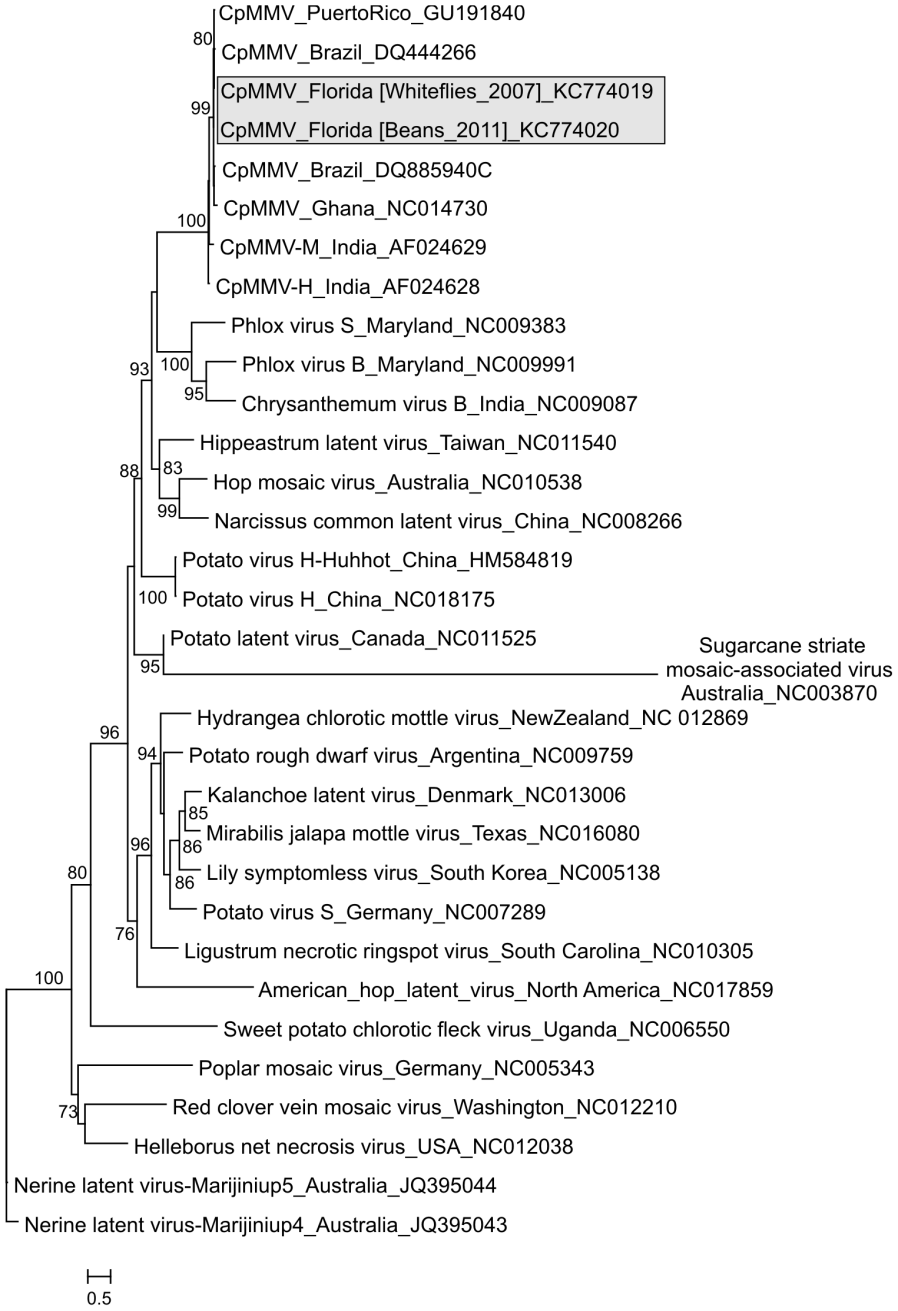
Maximum likelihood phylogenetic tree of capsid proteins (CP) found in different carlavirus species. Cowpea mild mottle virus (CpMMV) isolates identified in Florida, USA in this study are highlighted in grey. Branch support was assessed with the approximate likelihood ratio test and values >70% are shown.

PCR primers (Table S1) were used to obtain and sequence the entire CpMMV Florida genome from the field-collected whiteflies and each of the three bean plants experimentally infected with CpMMV Florida using whiteflies. The CpMMV genomes sequenced from bean plants, CpMMV Florida [Beans_2011] (Accession no. KC774020), are 100% identical to each other and share 99% genome-wide nucleotide identity with the genome retrieved from whiteflies collected four years earlier, CpMMV Florida [Whiteflies_2007] (Accession no. KC774019). The CpMMV Florida genomes exhibit organizations identical to members of the *Carlavirus* genus, including six open reading frames (ORFs) encoding the following proteins from 5′ to 3′: replication polyprotein, movement proteins [i.e., triple gene block (TGB)], capsid protein (CP) and nucleic acid binding (NB) protein (Fig. 1). Among the carlaviruses, the CpMMV Florida genomes are most closely related to the only other complete CpMMV genome sequence that was available at the time the analysis was performed, an isolate from Ghana (NC014730) [31], with which they share 67.5% genome-wide pairwise identity. Although the average amino acid pairwise identity for the complete protein complement of these two viral genomes is ∼62%, the CP exhibits 95% identity (Fig. 1). Phylogenetic analysis of the CP of different carlavirus species also supports identification of the Florida carlavirus as an isolate of CpMMV (Fig. 2). Based on pairwise distances among available CpMMV CP and NB sequences (Tables S3 and S4), the CpMMV Florida isolate may be more closely related to isolates from South America (Brazil) and the Caribbean (Puerto Rico) than to the Ghana isolate, since it shares 98–99% amino acid identity with these isolates.

Searches in the Pfam database using predicted amino acid sequences for each of the six ORFs present in the CpMMV Florida genomes revealed significant matches (e-value≪0.001) to conserved motifs observed in carlaviruses. The replication polyprotein contains four different domains characterized by viral methyltransferase [32], carlavirus endopeptidase (family C23 peptidase) [33], superfamily one helicase [34], and supergroup three RNA-dependent RNA polymerase [core motif: TGX_3_TX_3_NTX_22_GDD, where ‘X’ represents any amino acid residue] [35] motifs. Downstream from the replication polyprotein, there is a triple gene block involved in cell-to-cell movement with characteristic motifs of filamentous viruses, specifically the ‘potex-like’ class [36]. The first block contains NTPase/helicase sequence domains belonging to superfamily one helicases. The second and third genes contain the signature sequences GDX_6_GGXYXDG and CX_5_GX_8_C, respectively. Similar to several carlavirus species, the third gene in the TGB of the CpMMV Florida isolate lacks a standard start codon. The capsid protein exhibits both carlavirus- and potexvirus-specific domains and the carlavirus capsid signature sequence ‘GLGVPTE’ [17]. The putative NB protein encoded at the 3′ end of the CpMMV Florida genome, whose presence distinguishes virus species in the *Carlavirus* genus from other members of the *Betaflexiviridae* with similar genome organization (i.e., foveaviruses) [37], exhibits four characteristic cysteine residues in the pattern CX_2_CX_12_CX_4_C [38].

## Discussion

### Diversity of viruses identified in whiteflies

The VEM approach has been introduced as a strategy to survey viruses carried by insect vectors in a given region without *a priori* knowledge of the viral types present [13], [39]. Here VEM was used to detect both DNA and RNA plant viruses found in whiteflies collected from an experimental field station in Citra, FL. Strikingly, all of the DNA plant viruses identified with this deep Solexa sequencing effort were limited to the well-established whitefly-transmitted genus *Begomovirus* with high nucleotide identities (>88%) to known viral species. Although the short reads (maximum fragment size of 71 nt) hindered our ability to conclusively identify these begomoviruses to the species level even after assembly, results indicated that DNA viruses present in whiteflies from this site are dominated by members of a single genus. On the other hand, a diversity of RNA viral sequences from various families was detected and the VEM approach ultimately led to the discovery of the whitefly-transmitted carlavirus CpMMV in Florida, USA.

Since the VEM approach does not rely on sequence-specific primers/probes, this method should have recovered any type of DNA virus present in the whiteflies whose virions are resistant to chloroform and nuclease treatment. However, the use of a 0.22 µm filter during virus particle purification may have excluded larger non-plant DNA viruses which are commonly associated with insects (e.g., baculoviruses). The fact that only begomoviruses were identified suggests that this group indeed dominates the whitefly-transmitted DNA plant viruses, and perhaps exclusively occupies this niche. Future studies investigating plant DNA viruses in whiteflies from different locations using sequence-independent methods are needed to confirm whether or not whiteflies have evolved an exclusive relationship with begomoviruses.

In contrast to DNA viruses, the RNA library indicated that whiteflies from a single site can carry RNA viruses from disparate families. Currently, whiteflies are known to transmit four different groups of RNA viruses, including filamentous viruses from the Ipomovirus (*Potyviridae*), Crinivirus (*Closteoviridae*) and Carlavirus (*Betaflexiviridae*) genera, and icosahedral viruses from the *Torradovirus* genus *(Secoviridae*) [5]. Two of these groups were identified in the RNA metagenomic library, with high amino acid identities to known viruses including two criniviruses (Lettuce chlorosis virus (LCV) and Tomato chlorosis virus (TCV)) and a carlavirus (CpMMV). TCV has previously been reported from tomato in Florida [40]. However, this is the first evidence documenting the presence of CpMMV in the United States and LCV in the eastern United States. The remaining virus-like sequences identified in the RNA metagenomic library have low amino acid identities (<45%) with their top matches in the database. These novel viral sequences are most similar to groups that are not known to be carried by whiteflies and encompass divergent species, including viruses classified in three different families, as well as unclassified viruses (Table 2). It remains to be determined if these RNA viral sequences indeed represent novel whitefly-transmitted plant viruses, viruses infecting the whiteflies themselves, or simply transient viruses picked up by the whiteflies through feeding. Nevertheless, the detection of novel RNA viral sequences with weak similarities to known plant pathogens suggests that there are RNA plant viruses that have not yet been described.

### Discovery of the carlavirus CpMMV in Florida

The VEM approach led to the detection of the first whitefly-transmitted carlavirus (CpMMV Florida) in North America. This virus has been reported in a wide range of geographical areas including Africa, India, Asia, the Middle East, and South America, where it can infect and negatively impact important food crops [19]–[22], [41]–[43]. The host range of the CpMMV Florida isolate includes members of the *Chenopodeaceae* and *Fabaceae* families that have been previously reported as either natural or experimental hosts for CpMMV isolates from different regions. Many of the susceptible hosts did not show any visible signs of infection, which is similar to other CpMMV isolates [18], [21], [22], [44]. Asymptomatic infection by CpMMV may contribute to the high prevalence and transmission of this virus in some crop fields [18]. Although CpMMV Florida infects hosts that have been previously reported for CpMMV isolates from other regions, there are differences in the host ranges of isolates from different locations and crops. For example, CpMMV isolates from Israel and Ghana are able to infect representative members of the *Solanaceae* family [18], [45], whereas CpMMV Florida and isolates from Brazil, Thailand, and Southern Iran did not infect any members of this family [22], [41], [44]. Despite these differences, CpMMV isolates cannot be distinguished by electron microscopy or serologically, and no stringent comparative tests have been performed to determine if host range differences are sufficient to distinguish strains, pathotypes, or species [20].

A recent report published during the review process of this manuscript described six novel CpMMV isolates from Brazil that are closely related to CpMMV Florida (see below) [46]. Based on experimental hosts tested in both studies, most of the Brazilian isolates shared a similar host range with CpMMV Florida, mainly infecting members of the *Fabaceae*. However, two of the isolates (CpMMV∶BR∶BA∶02 and CpMMV∶BR∶GO∶01∶1) were able to infect a member of the *Solanaceae* (*Nicotiana glutinosa*) which CpMMV Florida fail to infect. CpMMV∶BR∶BA∶02 and CpMMV∶BR∶GO∶01∶1 share 98% and 93% genome-wide pairwise identity with CpMMV Florida, respectively. Therefore CpMMV isolates may exhibit different host ranges despite high nucleotide identities. Further research examining both the full genomes and experimental host range of all available CpMMV isolates will provide insight into which genetic differences explain differences in host range.

The CpMMV genome recovered from whiteflies collected in 2007 is 99% identical to the genome isolated from vegetation in the same field four years later. The genome organization of the CpMMV Florida isolate is similar to other carlaviruses. Full genome comparisons between CpMMV isolates from Florida and Ghana, which were the only genomes available at the time when the analyses were performed, clearly show that the CP shares a much greater degree of identity (95%) than non-structural proteins in the genomes (∼62%) (Fig. 1). This conservation of structural proteins but high variability in non-structural genes has been noted by other authors investigating CpMMV partial sequences [21], [31] and a recent report investigating six new CpMMV genomes from Brazil which share 93–99% genome-wide pairwise identity with the Florida isolate [46]. Despite the higher genetic distance among non-structural genes, CpMMV Florida exhibits all the core and functional domains that have been identified for these proteins. According to the ICTV classification criteria for members of *Betaflexiviridae* (formerly known as *Flexiviridae*), distinct species share <72% nucleotide or <80% amino acid identity between the entire CP or replication genes [47]. Due to the difference between the CP and non-structural identities, these criteria present a problem for properly classifying CpMMV isolates. Based on the replication gene, CpMMV Florida represents a novel species, whereas CP identities suggest it does not.

Unfortunately, most of the available CpMMV sequences only encompass the 3′ end of the genomes, containing the CP and/or NB since available carlavirus-specific degenerate PCR assays target this region [17], [48]. Most studies have based their classifications on ELISA, microscopy, and/or degenerate PCR targeting the coat protein and, thus, many viruses previously identified as CpMMV may actually represent different strains or even species. CP identities among available sequences only range from 88–99% whereas NB identities range from 56–99% (Tables S3 and S4). Furthermore, full genome comparisons between the Florida and Ghana isolates suggests that NB identities reflect identities for non-structural proteins (Fig. 1). Therefore our analysis suggests that the NB may be more representative of overall genomic similarities than the CP for classification purposes at the strain level. Based on this observation, it was expected that the CpMMV Florida isolate would be closely related to isolates from Brazil and Puerto Rico. This was confirmed with the recently released CpMMV genome sequences from Brazil which seem to belong to the same viral strain as CpMMV Florida. Due to the scarcity of genomic data regarding currently classified CpMMV isolates and strong CP similarities, we have named the Florida isolate as CpMMV; however, this classification may need to be revised as more genomic and infectivity data become available. It was also concluded that the Brazilian CpMMV isolates represented a new strain belonging to the same viral species as CpMMV based on their close phylogenetic relationship with the CpMMV isolate from Ghana. Interestingly, recombination analyses of CpMMV genomes from Brazil and Ghana suggested that low pairwise identities in the RdRP compared to the rest of the genome may be partly due to recombination events in this ORF [46]. Due to the occurrence of recombination events it may be necessary to use full genomic sequences for classification of carlavirus strains.

The biological significance of the highly conserved CP in CpMMV isolates is largely unknown; however, it may result from selective pressure of transmission driven by the whitefly vector. CpMMV isolates have been reported to be transmitted both non-persistently and semi-persistently as there are no latent periods for virus transmission in whiteflies but retention times vary from minutes to hours [20]. Regardless, even non-persistent transmission in other vector-virus systems can depend on specific interactions between vector and virus [49]. Therefore the diversity of CpMMV populations may be constrained by the need to retain specific interactions between its CP and the whitefly vector [8].

### Concluding remarks

The results of this study demonstrate that current understanding of RNA viruses found in *B. tabaci* whiteflies is not nearly as complete as that of DNA viruses, which appear to be restricted to the genus *Begomovirus*. Our findings indicate that the range of RNA viruses found in whiteflies may not be limited to the four groups that have been described since viral sequences with low amino acid identities likely represent novel groups. In addition to expanding current knowledge regarding viruses that can be captured by whiteflies, the VEM approach allowed us to expand the geographical range of CpMMV by documenting its presence in North America. Genomic comparisons among CpMMV genomes suggest that the classification criteria for carlaviruses need to be reevaluated, especially when considering variants that cannot be serologically distinguished. Future studies need to establish criteria to classify CpMMV variants and pathotypes by comparing genomic features, symptoms, infectivity and host range.

## Supporting Information

Table S1 **Primers used to amplify the CpMMV genome.**
(PDF)

Table S2 **Plant DNA viruses (i.e., begomoviruses) and associated satellite DNAs identified in whiteflies.**
(PDF)

Table S3 **Amino acid pairwise comparisons among available Cowpea mild mottle virus (CpMMV) capsid proteins (CP).**
(PDF)

Table S4 **Amino acid pairwise comparisons among available Cowpea mild mottle virus (CpMMV) nucleic acid binding (NB) proteins.**
(PDF)
