# Supplementary material for: RNA Viral Metagenome of Whiteflies Leads to the Discovery and Characterization of a Whitefly-Transmitted Carlavirus in North America
Source: PLoS One. 2014 Jan 21;9(1):e86748. doi: 10.1371/journal.pone.0086748 (PMC3897770; doi:10.1371/journal.pone.0086748)
Supplement: Table S1 — Primers used to amplify the CpMMV genome. (PDF) [file pone.0086748.s001.pdf]

Table S1. Primers used to amplify the CpMMV genome

Primer ID	Sequence (5' - 3')
FL-CpMMV_480R*	TTGAACTCCTAACAACAAAATCA
FL-CpMMV_480F	TGATTTTGTTGTTAGGAGTTCAA
FL-CpMMV_720R*	CCAACTAGCAACTCTGGTGGA
FL-CpMMV_720F	TCCACCAGAGTTGCTAGTTGG
FL-CpMMV_4820R	ACCTCATGTAGGGGGCAAAG
FL-CpMMV_3430F	ATCCTTGCCAAAGCGATTA
FL-CpMMV_4820R	ACCTCATGTAGGGGGCAAAG
FL-CpMMV_4680F	TAGATTCAGGGTAGCGAAGG
FL-CpMMV_5380R	TGGTTTCTTTAGCTATGCACATTC
FL-CpMMV_2990F	AAGGAAGGCAATTGTTGTGC
FL-CpMMV_6490R	CCCAGGTAAAGATGGTCGATT
FL-CpMMV_5390F	ACTTTCTGTGGGTGGCACTT
FL-CpMMV_dT-R	TTTTTTTTTTTTTTTTTAAAACCAGG

*Primers used for 5' RACE reaction
All PCR reactions contained 1 µl cDNA, 1 U Apex Red Taq Polymerase (Genesee), 1X NH4 buffer, 1.5 mM MgCL2,and 0.5 µM of each primer. Amplification was performed with an initial denaturation at 94ºC for 5 min, followed by 35 cycles of 94ºC for 45 sec, 50ºC for 45 min (incrementally decreasing the temperature by 0.1ºC on each cycle) , and 72ºC for 1.5 min, and a final extension at 72ºC for 8 min.
