# Supplementary material for: RNA Viral Metagenome of Whiteflies Leads to the Discovery and Characterization of a Whitefly-Transmitted Carlavirus in North America
Source: PLoS One. 2014 Jan 21;9(1):e86748. doi: 10.1371/journal.pone.0086748 (PMC3897770; doi:10.1371/journal.pone.0086748)
Supplement: Table S2 — Plant DNA viruses (i.e., begomoviruses) and associated satellite DNAs identified in whiteflies. (PDF) [file pone.0086748.s002.pdf]

Table S2. Plant DNA viruses (i.e., begomoviruses) and associated satellite DNAs identified in whiteflies.

Begomovirus or satellite match	Hits	Nt identity range (%)	Contig length (nt)
Unassigned begomovirus*	136	-	-
Sida golden mosaic virus	36	88-100	82-657
Cucurbit leaf curl virus	13	88-100	84-119
Tomato golden mottle virus	11	95-100	82-177
Euphorbia mosaic virus	5	95-100	84-107
Squash leaf curl virus	5	96-100	82-151
Whitefly VEM satellite	5	85-92	120-434
Bean mosaic crinkle Barbados virus	3	95-97	96-135
Okra mottle virus	3	100	97-109
Pepper golden mosaic virus	3	96-100	98-149
Sida yellow mosaic Yucatan virus	3	88-91	96-298
Abutilon mosaic virus-HW	2	89-95	135-138
Euphorbia yellow mosaic virus	2	93-97	82-95
Macroptilium golden mosaic virus	2	91-94	93-155
Macroptilium mosaic Puerto Rico virus	2	86-89	99-111
Potato yellow mosaic virus	2	86-95	125-154
Rhynchosia golden mosaic virus	2	89-90	82-126
Sida golden mosaic Florida virus-Malvastrum	2	94-100	83-95
Sida golden mottle virus	2	96-97	87-109
Tomato yellow leaf curl virus	2	100	83
Cabbage leal curl virus isolate Jamaica	1	89	84
Chino del tomate Amazonas virus	1	98	97
Corchorus yellow spot virus	1	90	242
Malvastrum yellow vein Baoshan virus	1	85	103
Okra yellow mottle Iguala virus	1	97	85
Rhynchosia golden mosaic Yucatan virus	1	93	118
Sida golden mosaic Honduras virus	1	87	175
Sida golden yellow vein virus	1	90	163
Sida micrantha mosaic virus	1	90	89
Tobacco leaf curl Cuba virus	1	100	121
Tobacco mottle leaf curl virus	1	92	86
Tobacco15 geminivirus	1	88	116
Tomato mottle virus	1	82	472
Tomato severe leaf curl virus	1	91	91
Tomato yellow vein streak virus	1	97	125
Wissadula golden mosaic virus	1	95	146

* Refers to sequences that had significant matches to more than one begomovirus with BLASTn scores within 10% of each other. Multiple matches make it impossible to assign these partial sequences.
