# Supplementary material for: RNA Viral Metagenome of Whiteflies Leads to the Discovery and Characterization of a Whitefly-Transmitted Carlavirus in North America
Source: PLoS One. 2014 Jan 21;9(1):e86748. doi: 10.1371/journal.pone.0086748 (PMC3897770; doi:10.1371/journal.pone.0086748)
Supplement: Table S3 — Amino acid pairwise comparisons among available Cowpea mild mottle virus (CpMMV) capsid proteins (CP). (PDF) [file pone.0086748.s003.pdf]

Table S3. Amino acid pairwise comparisons among available Cowpea mild mottle virus (CpMMV) capsid proteins (CP).

**CpMMV isolate**	Ghana	WF-FL	Bean-FL	B-Brazil	PR	Brazil	India-H	India-M	Taiwan	Taiwan
NC014730_CpMMV_Ghana	100									
CpMMV_Florida [Whiteflies 2007]	95.2	100								
CpMMV_Florida [Beans 2011]	95.6	99.6	100							
DQ885940_CpMMV_Barreiras (B)-Brazil	95.2	97.6	98.2	100						
GU191840_CpMMV_Puerto Rico (PR)	95.2	99.3	99.6	96.9	100					
DQ444266_CpMMV_Brazil	95.2	99	98.9	96.5	98.3	100				
AF024628_CPMMV-H_India	91.2	92.3	93	90.6	91.6	92	100			
AF024629_CPMMV-M_India	89.3	89.2	90.4	88.5	89.9	88.8	92.7	100		
JX070669_CpMMV_Taiwan	94.2	99	98.5	96.5	98.3	97.9	91.3	88.2	100	
JX020701_CpMMV-CY_Taiwan	95.3	98.3	98.9	97.9	97.6	97.2	92	89.2	97.9	100
