# Supplementary material for: RNA Viral Metagenome of Whiteflies Leads to the Discovery and Characterization of a Whitefly-Transmitted Carlavirus in North America
Source: PLoS One. 2014 Jan 21;9(1):e86748. doi: 10.1371/journal.pone.0086748 (PMC3897770; doi:10.1371/journal.pone.0086748)
Supplement: Table S4 — Amino acid pairwise comparisons among available Cowpea mild mottle virus (CpMMV) nucleic acid binding (NB) proteins. (PDF) [file pone.0086748.s004.pdf]

Table S4. Amino acid pairwise comparisons among available Cowpea mild mottle virus (CpMMV) nucleic acid binding (NB) proteins.

**CpMMV isolate**	Ghana	WF-FL	Bean-FL	B-Brazil	PR	Brazil	India-H	India-M	Venezuela
NC014730_CpMMV_Ghana	100								
CpMMV_Florida [Whiteflies 2007]	61.9	100							
CpMMV_Florida [Beans 2011]	61.9	98.1	100						
DQ885940_CpMMV_Barreiras (B)-Brazil	57.7	90.3	90.3	100					
GU191840_CpMMV_Puerto Rico (PR)	61.9	99	99	91.3	100				
DQ444266_CpMMV_Brazil	60.8	98.1	98.1	90.3	99	100			
AF024628_CPMMV-H_India	62.9	56.7	56.7	55.7	56.7	55.7	100		
AF024629_CPMMV-M_India	66	62.9	62.9	57.7	62.9	62.9	63.9	100	
JX310549_CpMMV_Venezuela	89.1	65.3	65.3	61.4	65.3	64.4	64	67	100
